# Looking beyond the mean: quantile regression for comparative physiologists

**DOI:** 10.1242/jeb.247122

**Published:** 2024-03-08

**Authors:** Coen Hird, Kaitlin E. Barham, Craig E. Franklin

**Affiliations:** School of the Environment, The University of Queensland, Brisbane (Magandjin), QLD 4072, Australia

**Keywords:** Median regression, Statistical toolbox, Ordinary least squares, Percentiles, Biostatistics

## Abstract

Statistical analyses that physiologists use to test hypotheses predominantly centre on means, but the tail ends of the response distribution can behave quite differently and underpin important scientific phenomena. We demonstrate that quantile regression (QR) offers a way to bypass some limitations of least squares regression (LSR) by building a picture of independent variable effects across the whole distribution of a dependent variable. We used LSR and QR with simulated and real datasets. With simulated data, LSR showed no change in the mean response but missed significant effects in the tails of the distribution found using QR. With real data, LSR showed a significant change in the mean response but missed a lack of response in the upper quantiles which was biologically revealing. Together, this highlights that QR can help to ask and answer more questions about variation in nature.

## Teaser

**Summary:** Comparative physiologists often focus on mean responses. Quantile regression is a tool that can help physiologists ask and answer more questions about nature by investigating more of the response distribution.

## INTRODUCTION

A fundamental assumption in biological sciences is that physiological systems are an emergent property of complex interactions between behavioural, morphological and biochemical pathways. Such complexity gives rise to considerable natural variation within and across physiological systems at multiple levels of organisation, which is at the intellectual core of comparative physiology (Somero, 2000). Exploration of variation by experimental biologists has shed light on a wealth of scientific phenomena explaining how life works and has uncovered many new questions that drive fascinating research agendas today. The contributions of comparative physiologists are increasingly useful and important in understanding the impacts of global change from molecular to ecosystem scales (Somero, 2011). Although there is a growing reframing of scientific thought towards the importance of variation and outliers in biological processes (Cook et al., 2021; Kar et al., 2021), most physiological studies address hypotheses about the central tendency of datasets rather than the nature of variation within them (Mykles et al., 2010). By solely focusing on means and averages, researchers can constrain their research questions and the answers they may find.

Least squares regression (LSR), one of the most widely used frequentist statistical approaches in empirical sciences, is a key component of systematic data analysis by physiologists. LSR techniques include ordinary least squares (OLS), multiple regression and ANOVA analyses. LSR has many perks: it is straightforward, parsimonious and describes relationships between dependent and independent variables well when assumptions are satisfied (but see Halsey and Perna, 2019). However, LSR is not without its drawbacks. Physiological datasets are often characterised by outliers, unequal variation (heteroscedasticity) and non-normal distributions – qualities that sometimes limit the utility of LSR. The application of LSR also shapes the way physiologists ask questions and formulate hypotheses. LSR techniques (such as OLS and ANOVA) predict the expected value (average) of the dependent variable for a given value of the independent variable. This is because LSR measures the deviation of data points away from the mean value. However, the relationship between variables based on the expected value of the response can look different to their relationship at other parts of the distribution. The application of standard LSR techniques in the analysis of scientific data is routine and normalised. This disposition of researchers to prioritise analytical methods that focus on central tendency has been called the ‘mean focus fallacy’ (Hohl, 2009), and can shape the generation of research questions to prioritise testing changes in the mean of the response. There is nothing inherently wrong with focusing on means, but not considering alternative tools which explore responses outside the centre of the distribution limits the scope of inquiry by excluding exploration of other areas of the sample distribution.

**Fig. 1.**
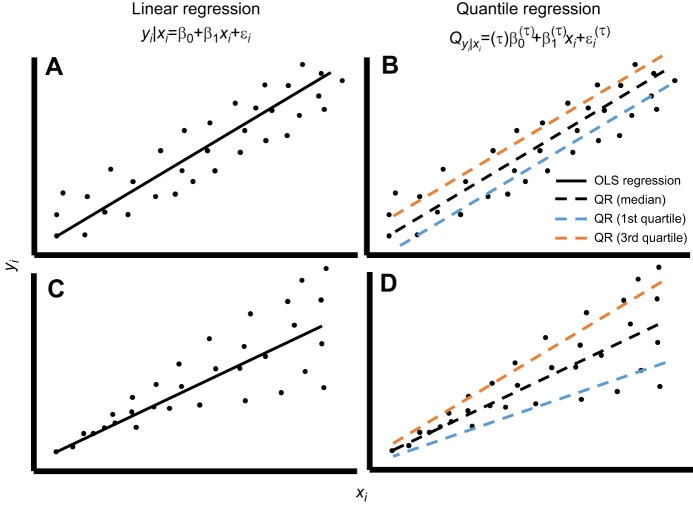
**Conceptual diagrams of models fitted using linear (LR) and quantile regressions (QR) for theoretical datasets with constant (top) and non-constant (bottom) variance.** LR and QR use much the same logic but unlike LR, QR does not rely on least squares and can estimate a response for any quantile of the data not just the mean. LR (A,C) models the expected value (mean) of *y_i_* with regression parameters β­_0_ (mean response when *x_i_*=0) and β_1_ (change in mean response with a single unit increase in *x_i_*), with error ε*_i_*. QR (B,D) estimates the conditional quantile *Q* of *y_i_* at specified quantiles τ of the distribution of *y_i_*, where regression coefficients and error are estimates at the τth quantile of *y_i_*. LR minimises the sum of squared error, whereas QR uses more involved mathematics to minimise the sum that gives penalties for over- or under-prediction. Quantile regression should be considered when: useful hypotheses and inferences can be made about values other than the mean outcome (i.e. responses in the tails of the distribution or effects of covariates on the entire distribution of *y_i_*); data are not normally distributed or have non-constant variance; or data have outliers which are biologically important that may skew linear regression. Quantile regression is also useful for monotonic transformations [such as log(*y_i_*)] because the means of log-transformed *y_i_* are equal to the log-transformed mean. This is not the case in LR, which calculates the geometric mean for back transformed *y_i_* when *x_i_* has not been log transformed. OLS, ordinary least squares.

Quantile regression (QR) is an emerging statistical methodology that goes beyond the limits of traditional LSR. QR estimates models of conditional quantile functions, or the relationship between independent variables and specified percentiles of a response (Fig. 1; Koenker, 2005; Yu et al., 2003), bypassing the need for normally distributed datasets without heteroscedasticity and outliers. QR has received attention in the field of economics (Huang et al., 2017; Koenker and Hallock, 2001) and ecology (Antúnez et al., 2023; Cade and Noon, 2003). However, QR is rarely used in comparative physiology, possibly because it is not well known or understood. QR offers researchers an opportunity to explore the nature of a response over the whole of its distribution (i.e. for a given quantile of the response). Imagine a research project concerned with understanding whether environmental temperature influenced the jumping distance of an adult amphibian. A researcher using LSR (i.e. ANOVA with cool and warm exposure treatments) that detected a statistically significant 2% reduction in mean jump distance for warm amphibians might conclude that temperature decreased jump distance. However, if a proportion of the amphibians in the warm treatment experienced a 15% increase in jump distance (i.e. in the 80th percentile), QR could describe this treatment effect which is otherwise overlooked or described qualitatively.

Here, we argue that QR analyses could be better applied by comparative physiologists to detect responses that other linear models would otherwise miss by prioritising investigation of the central tendency and focusing solely on changes in the mean. To illustrate this, we analysed a simple simulated dataset using traditional LSR and QR techniques. Then, we analysed real physiological data using the same methods. Our goal was not to provide a definitive guide on the mathematics and logic underpinning QR but to point comparative physiologists towards QR as a useful tool to search beyond the mean of physiological metrics when researching natural variation in the systems they study.

## MATERIALS AND METHODS

### Datasets

Two datasets were used for LSR and QR analyses: a simulated dataset and a real physiological dataset. The simulated dataset (*n*=200) was generated from the normal distribution for an arbitrary control (*n*=100; mean=50, s.d.=2) and treatment (*n*=100; mean=50, s.d.=7) group in R (v4.3.0; http://www.R-project.org/). The physiological dataset was obtained from an existing work that explored the effects of temperature on the amount of DNA damage incurred by amphibian larvae when exposed to ultraviolet radiation (UVR; see Hird et al., 2023). Only a subset of the physiological data (*n*=100) was used in the analyses presented here. In the current study, we focused on the concentration of DNA damage in whole tadpoles acclimated to 25°C (dependent variable) when exposed to UVR at 5 temperature (independent variable) levels (10, 15, 20, 25 and 30°C; *n*=20 larvae per acute temperature treatment).

### Statistical analyses

All analyses were conducted in the R statistical environment (v4.3.0; http://www.R-project.org/). Regression diagnostics were analysed using the performance package (v0.10.3; Lüdecke et al., 2021). Models were two-tailed and assumed a Gaussian error structure. α was at 0.05 for all tests.

For the simulated dataset, the response was compared between the arbitrary control and treatment group in an OLS regression that modelled the response as a function. The model met most assumptions of LSR except for equality of variance. Nine quantile regressions were fitted to the same data for the 10th to 90th percentile in 10% increments using the quantreg package (v5.95; https://CRAN.R-project.org/package=quantreg). Responses for OLS and QR were modelled using the categorical group as an independent dummy variable (with two levels: control or treatment). While QR is more often used with continuous response variables, we demonstrate here that it is reasonable to use QR for tests of comparison between categorical predictors because of the mathematically identical approaches of ANOVA and OLS. In both OLS and QR, standard errors for each quantile regression were constructed by bootstrapping (10,000 iterations).

For the DNA damage dataset, cyclobutene pyrimidine dimers (CPDs) formed from exposure to UVR were modelled using OLS regression with acute exposure temperature as the independent variable. CPDs were distributed non-normally so were log transformed prior to modelling with OLS regression. Assumptions were satisfied for the model. Nine quantile regressions with bootstrapped standard errors were computed as achieved using the simulated dataset above. DNA damage responses for OLS and QR were modelled using temperature as a continuous independent variable. Slope coefficients different from 0 represent a treatment effect at the conditional quantile. All bootstrapping was achieved using the *xy*-pair method.

## RESULTS AND DISCUSSION

**Fig. 2.**
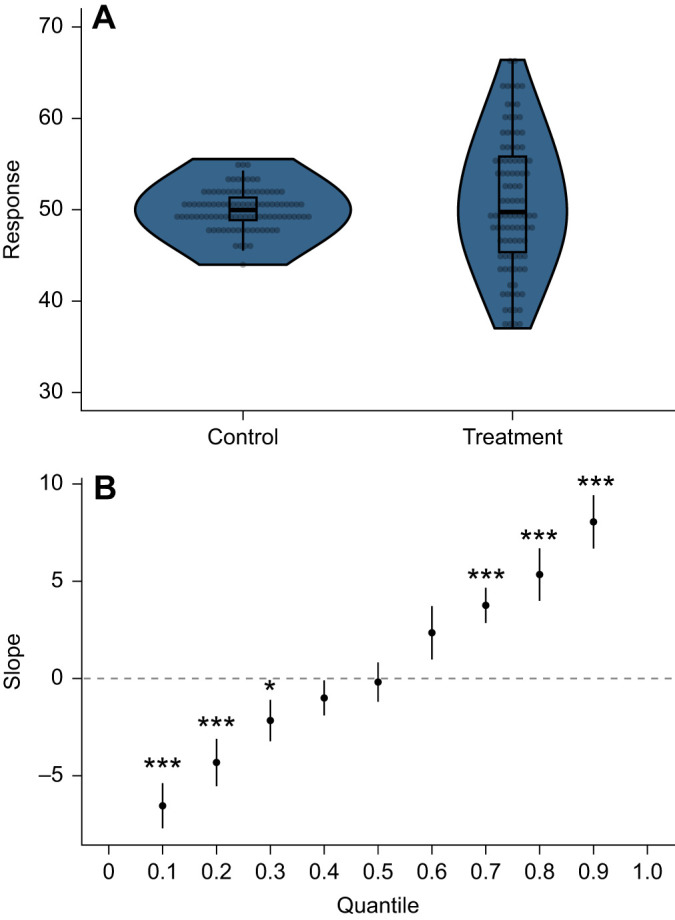
**The effect of treatment group on a response in a hypothetical dataset simulated from two different distributions with the same mean.** (A) Raw data (points), box plots and violin plots for both groups. (B) Slope coefficients for nine quantile regressions fitted for the 10th to 90th percentile in 10% increments, with bootstrapped 95% confidence intervals. Slope coefficients different from 0 represent a treatment effect at the conditional quantile. Asterisks represent statistically significant differences from 0 (*0.01<*P*<0.05; ***P<0.001). Quantile regression lines are not present in this diagram because of the presence of categorical predictors, so coefficients can only be estimated between the two points.

For the simulated dataset, there was no change in mean response between the two arbitrary groups using LSR (*t*_1,198_=0.54, *P*=0.59). QR showed no change in the response between treatment groups for the middle quantiles (0.4–0.6), an increase in the median treatment response for the upper quantiles (0.7–1.0) and a decrease in the median treatment response for the lower quantiles (0.1–0.3; Fig. 2).

**Fig. 3.**
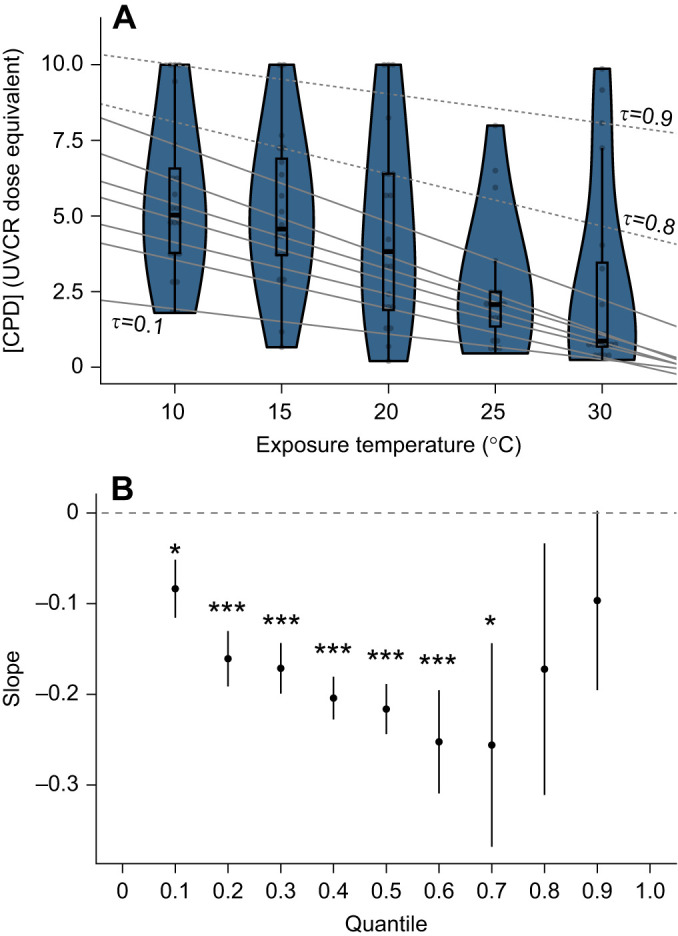
**The effect of exposure temperature on the accumulation of DNA damage in amphibian larvae following an acute high UV exposure.** (A) Raw data (points), box plots and violin plots of DNA damage [mapped using CPD detection and reported as units of ultraviolet-C radiation (UVCR)-dose equivalents] for each nominal level of exposure temperature. Lines are quantile (τ) regressions fitted for the 10th to 90th percentile in 10% increments, with the 10th, 80th and 90th quantile regression lines labelled. Solid lines represent slopes that were significantly different from 0 whereas dashed lines represent slopes that were not. (B) Plot of these slopes for each quantile regression with bootstrapped 95% confidence intervals. Asterisks represent statistically significant differences from 0 (*0.01<*P*<0.05; ***P<0.001).

For the physiological dataset, LSR showed that for every degree increase in temperature, the average (log) level of whole-animal DNA damage (UVC-irradiated equivalent) decreased by approximately −0.04 (*t*_1,30_=−5.48, *P*<0.001). Quantile regression revealed that the median CPD concentration in amphibian larvae decreased as temperature increased in the lower and middle of the distribution (0.1–0.7) but did not change in the upper quantiles (0.8–0.9; Fig. 3).

The results from the simulated data and the physiological dataset highlight two opposing possibilities of QR that tell different stories compared with the LSR outputs. For the simulated dataset, QR revealed a significant effect of treatment on the tails of the distribution of the response that was not considered when comparing the near-identical means. In the physiological dataset, QR regression showed significant decreases in DNA damage with increasing temperature for the 10th to 70th quantile, consistent with the findings of LSR that DNA damage from UVR reduces as temperature increases in amphibian larvae on average. However, the 80th to 90th percentile slopes were not significant. This suggests that temperature did not influence the level of DNA damage for larvae that incurred the most damage, because some larvae across all temperatures experienced extremely high levels of DNA damage. In other words, although OLS revealed that, on average, increased temperature decreased DNA damage, QR revealed that the highest levels of DNA damage were not associated with animals being in cooler temperatures. This finding of variation in the response itself raises interesting questions about what aspects of the biology of the high temperature, high DNA damage larvae were preventing them from undergoing a reduction in DNA damage that most of the larvae in this group experienced. Perhaps more importantly, approaching a statistical investigation with a bias toward solely analysing the mean may constrain researchers from generating interesting research questions and hypotheses that address questions and hypotheses concerned with differences around the upper and/or lower response. If there was evidence that a higher DNA damage level of 7.5 represented a lethal cutoff point, questions around whether temperature influences the capacity for maximal DNA damage following UVR exposure would be of critical importance.

For hypotheses concerning the central tendency of data, QR can be considered as a complementary or even alternative statistical tool to LSR. However, QR has important limitations that must be considered prior to its application. QR is unable to be used with binary dependent variables (Staffa et al., 2019). One of the biggest concerns with QR for experimental biology and comparative physiology is obtaining adequate statistical power to detect biologically relevant effects with low sample sizes. Low achieved statistical power due to sample size constraints is a pervasive issue across scientific disciplines and statistical methodologies (Button et al., 2013; Burgess et al., 2022). Many experimental designs in comparative physiology, for example, could not logistically hope to achieve a sample size of 100 as we used in this data simulation. QR estimates for data in the tails of the distribution, where there are generally fewer samples, create higher uncertainty than at central quantiles (Koenker and d'Orey, 1987; Staffa et al., 2019). While small sample size can also lead to underpowered LSR tests, it may disproportionately limit the analysis and interpretation of extreme quantiles in QR. When dealing with low sample sizes, it is recommended to use resampling techniques such as bootstrapping. It is possible that if the sample size is too small to bootstrap, both QR and LSR would not be adequately powered to detect significant effects. However, bootstrapped QR has shown an impressive capacity to handle the methodological difficulties of small samples (Koenker and Zhao, 1994; Cade and Richards, 2006; Tarr, 2012; Nikitina et al., 2019). Bootstrapping confidence around QR estimates also provides an important alternative to focusing on the *P*-value (Halsey, 2019; Halsey et al., 2015). While statistical software such as the quantreg package will provide bootstrapped estimates and standard errors for extreme percentiles (i.e. 0 and 100th percentiles), extreme care should be taken in trusting these estimates when the sample size is low.

While the examples modelled here were kept conceptually simple, QR is applicable to more complex models including non-linear forms with generalised additive models and splines (Koenker, 2005; Wei et al., 2019; Chabot et al., 2016). This is particularly useful in *in vivo* physiological datasets where many factors may contribute to variation in the response variable. QR has proven a useful modelling technique in studies of gene expression (Deng and Chowdhury, 2022), metabolism (Chabot et al., 2016; Sparling and Fedak, 2004), toxicity (Villain et al., 2016) and more (Baqué et al., 2015; Fleeger et al., 2010; McClain and Rex, 2001; Steluti et al., 2013; Yaniv et al., 2014). These and our own analyses illustrate critical and interrelated points around the drawbacks of focusing on the mean in physiological datasets. First, the variance of the response variable can change across an independent variable (homoscedasticity), which is not captured or explored using LSR. Second, there are important reasons that the tail ends of the response distribution can behave differently to the centre of the distribution in response to independent variables. Third, research questions of interest may include or directly relate to quantiles, rather than means of physiological data. And fourth, QR is a useful statistical method to describe relationships within physiological datasets instead of or in addition to LSR. Ultimately, the inclusion of QR in the comparative physiologist's statistical toolbox can help to ask and answer more questions about variation in the natural world.

## ECR Spotlight

This article has an associated ECR Spotlight interview with Coen Hird.

## References

[JEB247122C1] Antúnez, P., Wehenkel, C., Hernández-Díaz, J. C. and Garza-López, M. (2023). Quantile regression as a complementary tool for modelling biological data with high variability. *J. Trop. For. Sci.* 35, 130-140. 10.26525/jtfs2023.35.2.130

[JEB247122C2] Baqué, M., Filmann, N., Verhoff, M. A. and Amendt, J. (2015). Establishment of developmental charts for the larvae of the blow fly *Calliphora vicina* using quantile regression. *Forensic Sci. Int.* 248, 1-9. 10.1016/j.forsciint.2014.12.020 25590766

[JEB247122C3] Burgess, B. J., Jackson, M. C. and Murrell, D. J. (2022). Are experiment sample sizes adequate to detect biologically important interactions between multiple stressors? *Ecol. Evol.* 12, e9289. 10.1002/ece3.9289 36177120 PMC9475135

[JEB247122C4] Button, K. S., Ioannidis, J. P. A., Mokrysz, C., Nosek, B. A., Flint, J., Robinson, E. S. J. and Munafò, M. R. (2013). Power failure: why small sample size undermines the reliability of neuroscience. *Nat. Rev. Neurosci.* 14, 365-376. 10.1038/nrn3475 23571845

[JEB247122C5] Cade, B. S. and Noon, B. R. (2003). A gentle introduction to quantile regression for ecologists. *Front. Ecol. Environ.* 1, 412-420. 10.1890/1540-9295(2003)001[0412:AGITQR]2.0.CO;2

[JEB247122C6] Cade, B. S. and Richards, J. D. (2006). A permutation test for quantile regression. *J. Agric. Biol. Environ. Stat.* 11, 106-126. 10.1198/108571106X96835

[JEB247122C7] Chabot, D., Koenker, R. and Farrell, A. P. (2016). The measurement of specific dynamic action in fishes. *J. Fish Biol.* 88, 152-172. 10.1111/jfb.12836 26768974

[JEB247122C8] Cook, C. N., Freeman, A. R., Liao, J. C. and Mangiamele, L. A. (2021). The philosophy of outliers: reintegrating rare events into biological science. *Integr. Comp. Biol.* 61, 2191-2198. 10.1093/icb/icab166 PMC9076997 34283241

[JEB247122C9] Deng, D. and Chowdhury, M. H. (2022). Quantile regression approach for analyzing similarity of gene expressions under multiple biological conditions. *Stats* 5, 583-605. 10.3390/stats5030036

[JEB247122C10] Fleeger, J. W., Johnson, D. S., Carman, K. R., Weisenhorn, P. B., Gabriele, A., Thistle, D. and Barry, J. P. (2010). The response of nematodes to deep-sea CO_2_ sequestration: a quantile regression approach. *Deep Sea Res. Part I Oceanogr. Res. Pap.* 57, 696-707. 10.1016/j.dsr.2010.03.003

[JEB247122C11] Halsey, L. G. (2019). The reign of the p=value is over: what alternative analyses could we employ to fill the power vacuum? *Biol. Lett.* 15, 20190174. 10.1098/rsbl.2019.0174 31113309 PMC6548726

[JEB247122C12] Halsey, L. G. and Perna, A. (2019). Regression dilution in energy management patterns. *J. Exp. Biol.* 222, jeb197434. 10.1242/jeb.197434 30833460

[JEB247122C13] Halsey, L. G., Curran-Everett, D. C., Vowler, S. L. and Drummond, G. B. (2015). The fickle P value generates irreproducible results. *Nat. Methods* 12, 179-185. 10.1038/nmeth.3288 25719825

[JEB247122C14] Hird, C., Cramp, R. L. and Franklin, C. E. (2023). Thermal compensation reduces DNA damage from UV radiation. *J. Therm. Biol.* 117, 103711. 10.1016/j.jtherbio.2023.103711 37717403

[JEB247122C15] Hohl, K. (2009). Beyond the average case: the mean focus fallacy of standard linear regression and the use of quantile regression for the social sciences. Available at SSRN: 10.2139/ssrn.1434418

[JEB247122C16] Huang, Q., Zhang, H., Chen, J. and He, M. (2017). Quantile regression models and their applications: a review. *J. Biom. Biostat.* 8, 1000354. 10.4172/2155-6180.1000354

[JEB247122C17] Kar, F., Nakagawa, S., Friesen, C. R. and Noble, D. W. A. (2021). Individual variation in thermal plasticity and its impact on mass-scaling. *Oikos* 130, 1131-1142. 10.1111/oik.08122

[JEB247122C18] Koenker, R. (2005). *Quantile Regression*. Cambridge University Press.

[JEB247122C19] Koenker, R. and d'Orey, V. (1987). Computing regression quantiles. *J. R. Stat. Soc. Ser. C. Appl. Stat.* 36, 383-393. 10.2307/2347802

[JEB247122C20] Koenker, R. and Hallock, K. F. (2001). Quantile regression. *J. Econ. Perspect.* 15, 143-156. 10.1257/jep.15.4.143

[JEB247122C21] Koenker, R. and Zhao, Q. (1994). L-estimation for linear heteroscedastic models. *J. Nonparametr. Stat.* 3, 223-235. 10.1080/10485259408832584

[JEB247122C22] Lüdecke, D., Ben-Shachar, M. S., Patil, I., Waggoner, P. and Makowski, D. (2021). performance: an R package for assessment, comparison and testing of statistical models. *J. Open Source Softw.* 6, 3139. 10.21105/joss.03139

[JEB247122C23] McClain, C. and Rex, M. (2001). The relationship between dissolved oxygen concentration and maximum size in deep-sea turrid gastropods: an application of quantile regression. *Mar. Biol.* 139, 681-685. 10.1007/s002270100617

[JEB247122C24] Mykles, D. L., Ghalambor, C. K., Stillman, J. H. and Tomanek, L. (2010). Grand challenges in comparative physiology: integration across disciplines and across levels of biological organisation. *Integr. Comp. Biol.* 50, 6-16. 10.1093/icb/icq015 21558183

[JEB247122C25] Nikitina, L., Paidi, R. and Furuoka, F. (2019). Using bootstrapped quantile regression analysis for small sample research in applied linguistics: some methodological considerations. *PLoS One* 14, e0210668. 10.1371/journal.pone.0210668 30640925 PMC6331127

[JEB247122C26] Somero, G. N. (2000). Unity in diversity: a perspective on the methods, contributions, and future of comparative physiology. *Annu. Rev. Physiol.* 62, 927-937. 10.1146/annurev.physiol.62.1.927 10845117

[JEB247122C27] Somero, G. N. (2011). Comparative physiology: a “crystal ball” for predicting consequences of global change. *Am. J. Physiol. Regul. Integr. Comp. Physiol.* 301, R1-R14. 10.1152/ajpregu.00719.2010 21430078

[JEB247122C28] Sparling, C. E. and Fedak, M. A. (2004). Metabolic rates of captive grey seals during voluntary diving. *J. Exp. Biol.* 207, 1615-1624. 10.1242/jeb.00952 15073194

[JEB247122C29] Staffa, S. J., Kohane, D. S. and Zurakowski, D. (2019). Quantile regression and its applications: a primer for anesthesiologists. *Anesth. Analg.* 128, 820-830. 10.1213/ANE.0000000000004017 30649075

[JEB247122C30] Steluti, J., Verly, E. Jr., Fisberg, R. M. and Marchioni, D. M. L. (2013). The effect of fruits and vegetables in the elevated plasma homocysteine: experience of using a quantile regression approach. *FASEB J.* 27, 1077.15. 10.1096/fasebj.27.1_supplement.1077.15 PMC4218785 25365261

[JEB247122C31] Tarr, G. (2012). Small sample performance of quantile regression confidence intervals. *J. Stat. Comput. Simul.* 82, 81-94. 10.1080/00949655.2010.527844

[JEB247122C32] Villain, J., Minguez, L., Halm-Lemeille, M., Durrieu, G. and Bureau, R. (2016). Acute toxicities of pharmaceuticals toward green algae. Mode of action, biopharmaceutical drug disposition classification system and quantile regression models. *Ecotoxicol. Environ. Saf.* 124, 337-343. 10.1016/j.ecoenv.2015.11.009 26590695

[JEB247122C33] Wei, Y., Kehm, R. D., Goldberg, M. and Terry, M. B. (2019). Applications for quantile regression in epidemiology. *Curr. Epidemiol. Rep.* 6, 191-199. 10.1007/s40471-019-00204-6

[JEB247122C34] Yaniv, S., Elad, D. and Holzman, R. (2014). Suction feeding across fish life stages: flow dynamics from larvae to adults and implications for prey capture. *J. Exp. Biol.* 217, 3748-3757. 10.1242/jeb.104331 25189373

[JEB247122C35] Yu, K., Lu, Z. and Stander, J. (2003). Quantile regression: applications and current research areas. *J. R. Stat. Soc. Ser. D Stat.* 52, 331-350. 10.1111/1467-9884.00363

